# Verification of genetic differences and immune cell infiltration subtypes in the neuroblastoma tumour microenvironment during immunotherapy

**DOI:** 10.1186/s12957-022-02641-y

**Published:** 2022-05-28

**Authors:** Bo Qian, Jing Sun, Pengcheng Zuo, Min Da, Xuming Mo, Yongjun Fang

**Affiliations:** 1 Department of Cardiothoracic Surgery, Children’s Hospital of Nanjing Medical University, 72 Guangzhou Road, Nanjing, 210008 China; 2 Department of Hematology and Oncology, Children’s Hospital of Nanjing Medical University, Nanjing, China

**Keywords:** Neuroblastoma, Immune cell infiltration, Immunotherapy, Biomarker, Prognosis

## Abstract

**Background:**

Improved understanding of the tumour microenvironment (TME) has enabled remarkable advancements in research on cancer progression in the past few years. It is crucial to understand the nature and function of the TME because precise treatment strategies, including immunotherapy, for managing specific cancers have received widespread attention. The immune infiltrative profiles of neuroblastoma (NB) have not yet been completely illustrated. The purpose of this research was to analyse tumour immune cell infiltration (ICI) in the microenvironment of NB.

**Methods:**

We applied the CIBERSORT and ESTIMATE algorithms to evaluate the ICI status of 438 NB samples. Three ICI models were selected, and ICI scores were acquired. Subgroups with high ICI scores determined based on the presence of immune activation signalling pathways had better overall survival.

**Results:**

Genes involved in the immunosuppressive heparan sulphate glycosaminoglycan biosynthesis signalling pathway were markedly enriched in the low ICI score subgroup. It was inferred that patients with high ICI NB subtypes were more likely to respond to immunotherapy and have a better prognosis than those of patients with low ICI NB subtypes.

**Conclusion:**

Notably, our ICI data not only provide a new clinical and theoretical basis for mining NB prognostic markers related to the microenvironment but also offer new ideas for the development of NB precision immunotherapy methods.

## Introduction

Neuroblastoma (NB) is an extracranial malignant solid tumour derived from embryonic neural crest cells, and it accounts for the mortality of nearly 15% of all paediatric tumour patients [1, 2]. The age of onset of NB is usually early, and the median age at diagnosis is 17 months. It usually occurs in the adrenal medulla or paravertebral ganglia, so it can be located in the abdominal cavity, thoracic cavity, neck, etc. Approximately half of NB patients have stage distant metastases at the first diagnosis [3]. Although the 5-year survival rate of low-risk NB is greater than 95%, the 5-year survival rate of high-risk NB is still less than 50% after comprehensive treatment, such as surgery, radiotherapy, chemotherapy, and biological therapy, because of its later recurrence, distant metastasis, and drug resistance [4].

Over the last decades, various immunotherapies have been applied to treat an increasing number of refractory malignant cancers [5]. Immunotherapy methods enabling neuroblastoma treatment have been expected to become the most promising treatment strategy and to provide a new direction for its treatment. However, immunotherapeutic strategies benefit only a subset of patients, while most patients face the problem of resistance to immunotherapy [6]. With advanced knowledge about the immune system and tumour microenvironment, new targets may facilitate immunotherapy treatment for NB patients.

High-risk NB has a high degree of malignancy and is resistant to radiotherapy and chemotherapy. Therefore, new treatment strategies are needed to improve their biological conditions. The tumour-specific antigen and immune microenvironment phenotype may be used as predictive indicators for evaluating the effect of immunotherapy. The tumour microenvironment (TME) is a dynamic system formed by the interaction of cancer cells with tumour interstitial cells, immune cells, extracellular matrix, surrounding vascular tissues, and signalling molecules [7]. In addition to the invasion, migration, and proliferation of tumour cells, the occurrence of tumour metastasis is also closely related to its microenvironment. The inhibitory phenotype and heterogeneity of the TME are important factors that promote tumour progression and affect immune efficacy [8, 9]. Due to multiple factors, such as an immunosuppressive microenvironment and a lack of tumour-specific antigen expression, high-risk NB tumours are not sensitive to immunotherapy and belong to the cold tumour category, while low-risk NB tumours have the features of hot tumours. Increased expression of tumour-associated M2 macrophages (CD163 +) could decrease the function of NK cells, DCs, and T cells in these cold tumours [10]. The main types of infiltrating stromal cells are macrophages and fibroblasts. The cell-to-cell interaction between tumour-associated M2 macrophages and cancer-associated fibroblasts promotes the progression of neuroblastoma by mutual induction of recruitment and activation. Studies have confirmed that these cells exhibit tumour-promoting activity by activating the ERK1/2 and STAT3 signalling pathways in neuroblastoma cells [11]. These cells promote the proliferation of NB cells and secrete a variety of inflammatory cytokines and chemokines, such as IL-6, IL-8, VEGF-A, CCL-2, and CXCL12, to enhance resistance to chemotherapy. Disialoganglioside (GD2) is highly expressed in NB cells, resulting in impaired immune function of T cells and immunotherapy resistance [12]. Programmed death 1 (PD-1), the main immune checkpoint receptor, is expressed on human T cells, dendritic cells, and natural killer T cells, while programmed death ligand 1 (PD-L1) is expressed on the surface of NB cells. The PD-1/PD-L1 pathway inhibits the proliferation and differentiation of T cells after PD-1 binds to PD-L1 on NB cells and then induces activated T cells to transform into inactive T cells or undergo apoptosis [13, 14].

Next-generation sequencing (NGS) has become an important aspect of precise tumour diagnosis and treatment that is used for multiple purposes, such as the detection of driver genes related to tumour-targeted therapy, the analysis of drug resistance mechanisms, the evaluation of cancer metastasis and prognosis, and the full dynamic monitoring of tumour patients. CIBERSORT is a tool that uses a deconvolution algorithm to predict the proportion of cells. It can analyse the cellular composition of immune tissues based on standardized expression data and measure the abundance of specific cell types [15, 16]. We hypothesized that the immune resistance of most neuroblastoma patients was closely related to the characteristics of immune cell infiltration. To explore whether the immune cell infiltration score can be a neuroblastoma-specific prognostic biomarker and develop new ideas for immunotherapy methods, we explored the gene expression data of TARGET-NB and GSE85047 to obtain a comprehensive overview of tumour immunity by two algorithms (CIBERSORT and ESTIMATE) [17]. Then, we divided NB into different subtypes by the type of immune cell infiltration [18]. Establishing the ICI score as a method for characterizing various immune features could identify NB patients who respond to immunotherapy.

## Methods

### Neuroblastoma data collection

The RNA data (level 3) were downloaded from the Therapeutically Applicable Research to Generate Effective Treatments (TARGET)-NB database (https://ocg.cancer.gov/programs/target/data-matrix). The Affymetrix microarray datasets GSE85047 were obtained from the Gene Expression Omnibus (GEO, http://www.ncbi.nlm.nih.gov/geo) database. Clinical data such as age, INSS, survival and outcome were also downloaded from TARGET (155 samples) and GEO (283 samples). Fragments per kilobase of transcripts per million (FPKM) data from the TARGET samples were converted to transcripts per million (TPM). The gene mRNA expression data and clinical characteristics from the TARGET and GEO databases are publicly available and open to access, so this study did not need the approval from the ethics committee. To obtain key information from these expression matrices, Perl scripts and R software (R-project.org) were used to merge and preprocess the original data. If the gene symbol corresponded to multiple probe IDs, the average value of the probes was calculated as the expression level of the gene. Censored data were removed, and data from patients with no detailed information on overall survival time were removed. After rigorous screening, a total of 438 samples were included in the study.

### Immune score determination for the NB microenvironment and immune cellular fraction estimates

We calculated the immune score of NB patients with the ESTIMATE algorithm. All patients were classified into a high group and a low group according to their immune/matrix scores. The CIBERSORT algorithm was used to evaluate tumour infiltrating immune cell (TIIC) data in NB samples from the TARGET and GEO cohorts [19, 20]. Visualization of the results was completed using the R package. The hierarchical agglomerative clustering of NB was performed according to the types of ICI of the patients. This analysis was executed using the R package and repeated 1000 times to ensure the stability of the classification.

### Differentially expressed genes (DEGs) related to the ICI clusters

Patients were classified into different ICI clusters based on immune cell infiltration. Absolute fold changes > 1 and *p* < 0.05 (after adjustment) were determined as the cut-off criteria for DEGs. Data were analysed by the package edge R [18].

### Pathway and function enrichment analysis

Functional enrichment analysis of immune cell infiltration-related DEGs was performed with the “clusterProfiler” R package to identify the Gene Ontology (GO) and Kyoto Encyclopedia of Genes and Genomes (KEGG) terms. A *P* value < 0.05 was considered the cut-off value [21].

### Principal component analysis (PCA)

PCA is a statistical method that applies the idea of dimensionality reduction to transform multiple variables into a few uncorrelated comprehensive variables [22]. We performed PCA to define the differences in infiltrating immune cells among distinct groups. The PCA diagram could be used classify different infiltrating immune cells as variables to describe the differences in NB samples.

### Statistical analyses

All statistical analyses were performed using SPSS 22.0 software. Kaplan–Meier analysis was conducted to calculate the survival curve of the subgroups in the dataset. The log-rank test was used to assess statistically significant differences. The Wilcoxon test was performed to compare the two groups, while the Kruskal–Wallis test was applied to compare more than two groups.

## Results

### The profile of immune cell infiltration in the TME of NB samples

**Fig. 1:**
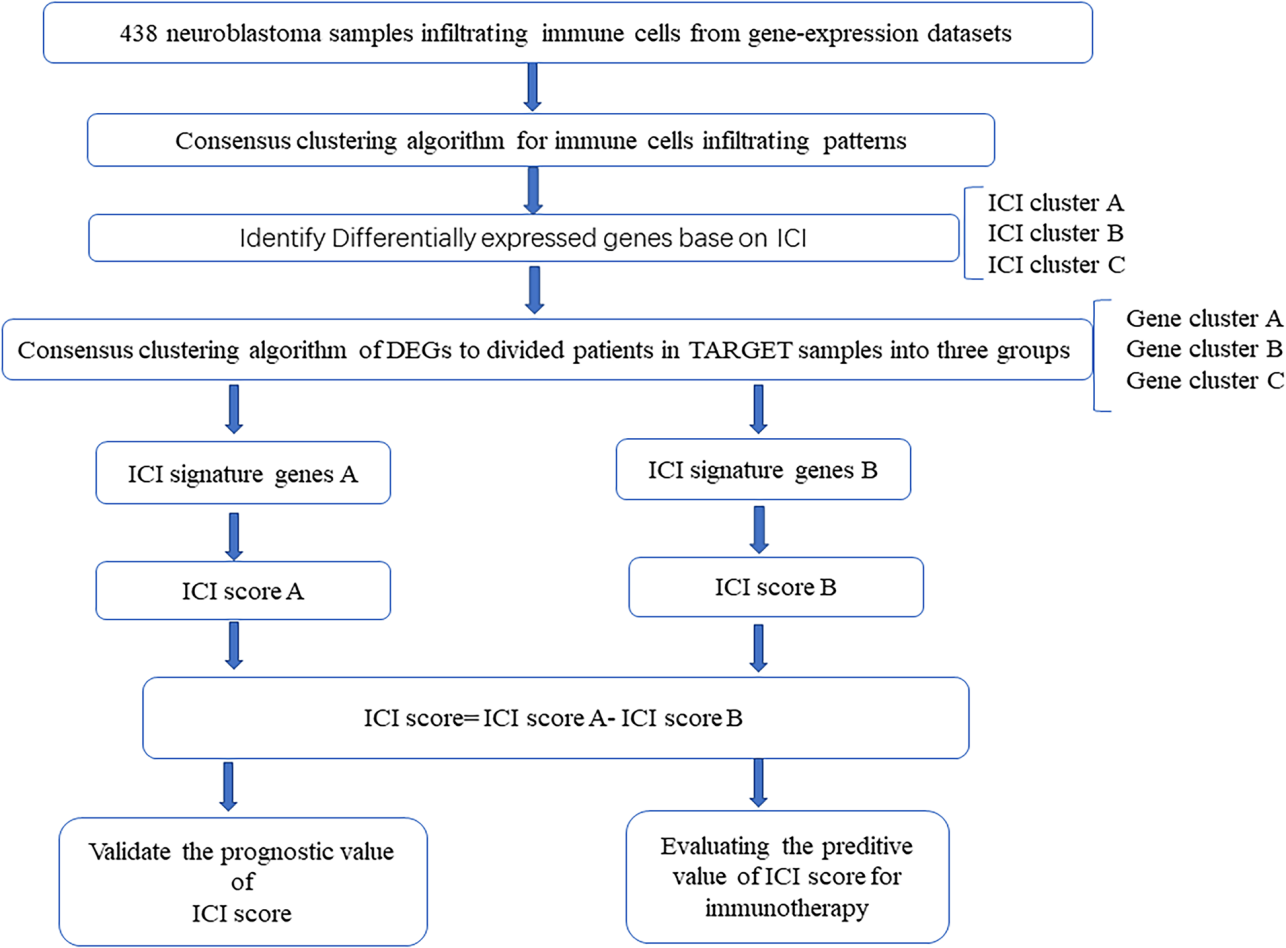
Summary of study design

The study design is shown in Fig. 1. In our study, we first adopted two algorithms (ESTIMATE and CIBERSORT) to analyse immune cell subtypes in NB samples with the R platform. Subsequently, the ConsusClusterPlus package was used to perform unsupervised clustering of 438 NB samples from GSE85047 and (TARGET)-NB to define different subgroups.

**Fig. 2:**
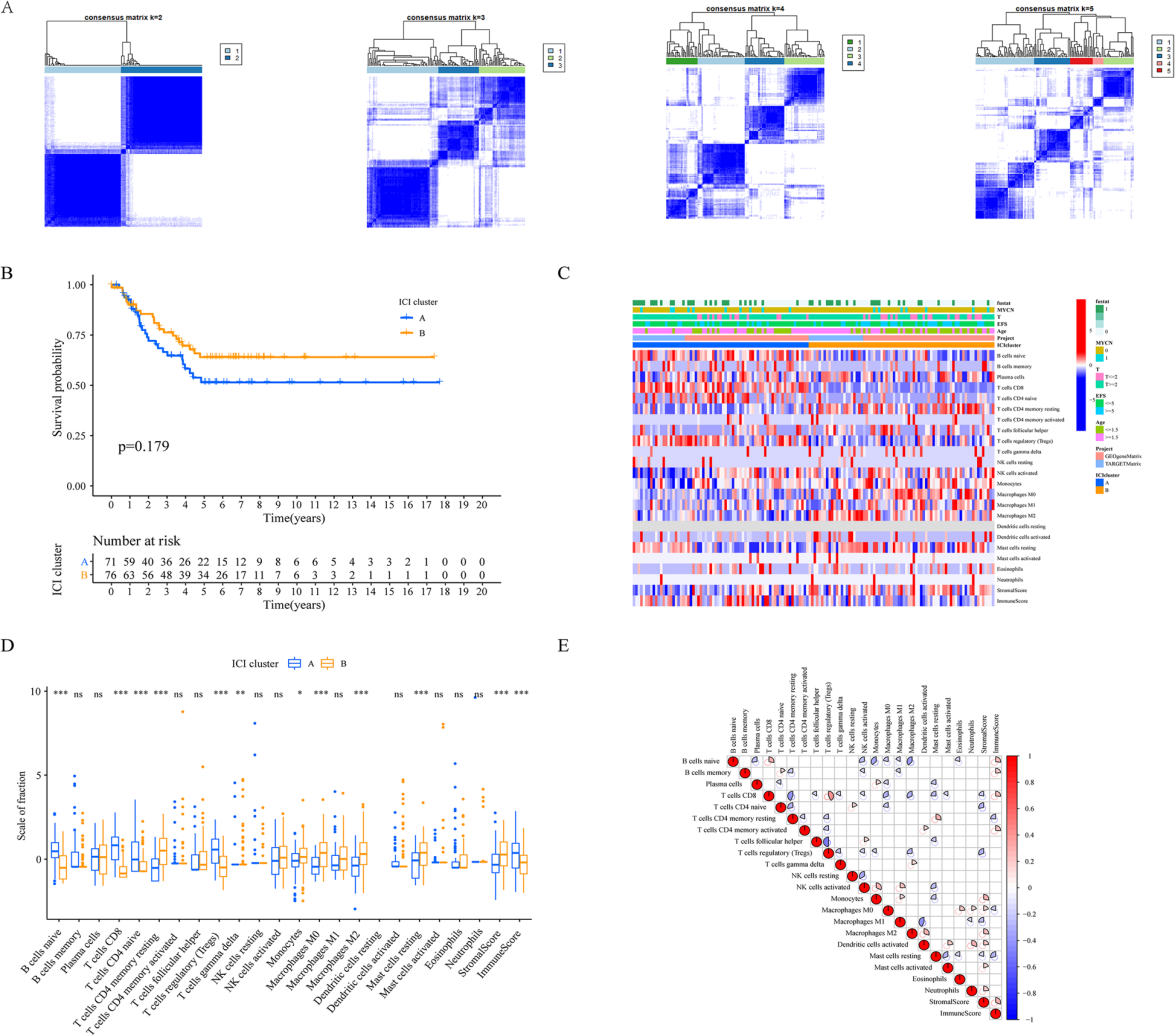
The profiles of immune cell infiltration in the TME of NB. **A** 1000 hierarchical clustering was performed to determine the stable *k* value of the consensus matrix for all NB patients (*k* = 2–5). **B** Association between tumour-associated immune cells in NB patients and overall survival (*p* = 0. 179). **C** The distribution of infiltrating immune cells in different NB groups. **D** The Kruskal–Wallis test was used to test the differences in infiltrating immune cells, immune scores and matrix scores in different ICI clusters. NS no significance; ***p < 0.001; **p < 0.01; *p < 0.05. **E** The interaction of tumour-related infiltrating immune cells in NB

The Kaplan–Meier curves indicated that ICI A and ICI B were not significantly associated with improved outcome (log rank test, *p* = 0.179; Fig. 2 A–C). Then, we assessed the differences in immune cell subtype between the two ICI groups using the R platform.

The relative content of resting dendritic cells in all samples was nearly 0. Among the two main immune subtype groups, the immune cells found in ICI Cluster A had a high ImmuneScore value and mainly comprised naive B cells, regulatory T cells (Tregs), naive CD4 T cells and CD8 T cells, while the samples in ICI Cluster B were represented by a significantly higher StromalScore and significantly higher densities of resting memory CD4 T cells, M0 macrophages, M2 macrophages and resting mast cells (Fig. 2D). In addition, the correlation coefficients of the NB TME were analysed and visualised using a heat map (Fig. 2E). We found that the relative content of M2 cells in NB samples was significantly negatively correlated with those of several cell types (CD8 T cells, naive B cells, and regulatory T cells).

### Identified immune gene subtype

**Fig. 3:**
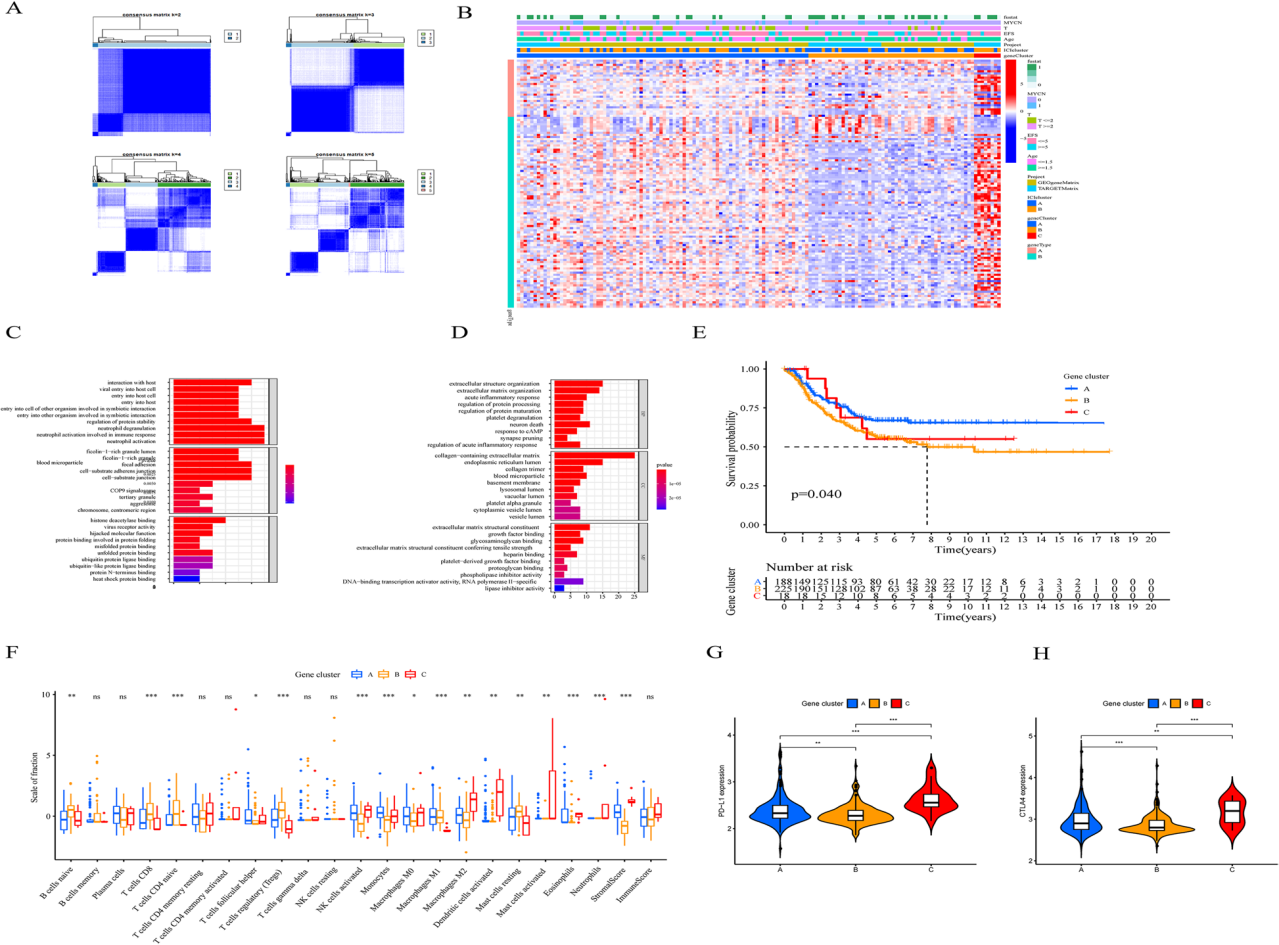
Distinguishing immune-related gene subtypes. **A** The DEGs in different groups were divided into three groups by unsupervised cluster analysis as gene Clusters A–C. **B** The distribution of infiltrating immune cells in different NB subtypes. **C**, **D** Gene ontology (GO) enrichment analysis of notable genes of different ICI subtypes: ICI notable genes. **E** Association between different NB subgroups and overall survival, *p* = 0.04. **F** Differences in infiltrating immune cells, immune scores and matrix scores of different NB gene subtypes. NS, no significance; ***p < 0.001; **p < 0.01; *p < 0.05. **G**, **H** The distribution of CTLA4 and PD-L1 expression among different ICI genotypes (*p* < 0.001)

After the gene datasets from TARGET-NBL and GSE85047 samples were integrated with R (version 3.6.1), we identified an aggregate of 117 DEGs by the limma package. Then, we divided these samples into three groups based on the DEGs and called them gene Clusters A-C (Fig. 3A). There were 24 DEGs significantly positively related to the gene cluster that were named ICI gene features A, while the remaining DEGs were called ICI gene features B. Dimension reduction was used to reduce interference by the Boruta algorithm. A heatmap of the 117 DEGs was drawn in R software to show the correlation between the genomic traits and clinical features (Fig. 3B) [23]. The results of the enrichment analyses of the GO terms and KEGG pathways are shown in Fig. 3C and D. The ICI feature gene A group were involved in pathways including neutrophil activation involved in the immune response, ficolin − 1 − rich granule production and neutrophil degranulation, while the ICI feature gene B group was associated with extracellular matrix structural constituent, collagen-containing extracellular matrix and extracellular structure organization pathways.

In addition, the Survival and survminer packages of R software were used to explore the relationship between survival features and ICI gene clusters. The Kaplan–Meier analysis revealed that the samples in gene Cluster A were significantly associated with improved outcome, while samples in the remaining gene clusters were associated with a poor prognosis (log rank test, *p* = 0.040; Fig. 3E). Gene Cluster C samples were characterized by more abundant stromal cell infiltration and immunosuppression-related M2 macrophages, suggesting that the result of immunotherapy was unfavourable [24, 25]. The expression levels of PD-L1 and CTLA4 were significantly different in these three genome clusters (Kruskal–Wallis, *p* < 0.001; Fig. 2G and H). Across the three different ICI gene Clusters, CTLA4 and PD-L1 had the lowest expression levels in ICI gene Cluster B.

### Calculation of the ICI score

**Fig. 4:**
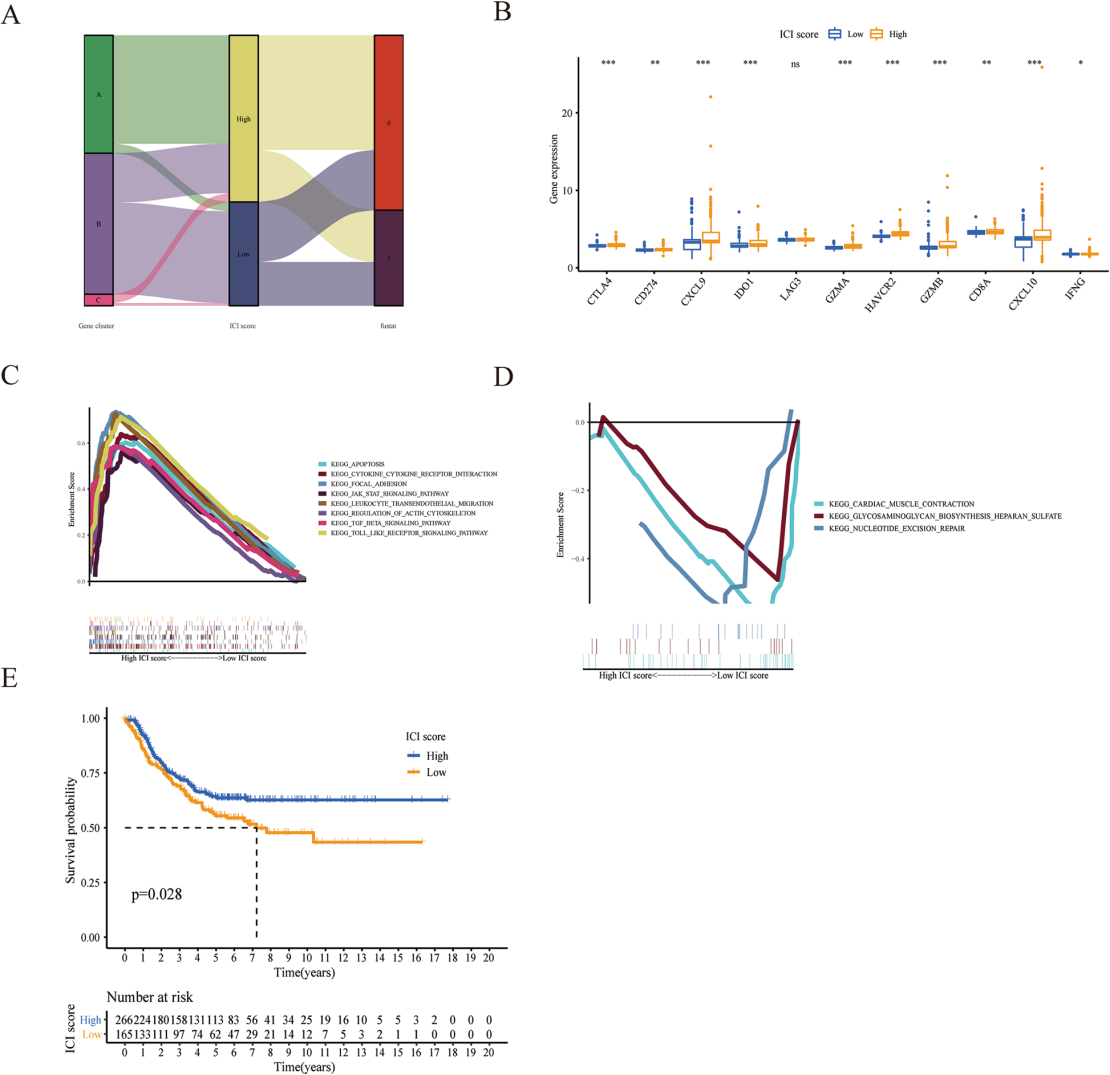
Establishment of ICI score. **A** Distribution of the alluvial graph of different ICI clusters, ICI scores and survival statuses. **B** The expression of immune checkpoint-related targets and immune activation-related targets in different ICI score subgroups. **C** The main enrichment analysis result in the low ICI score subgroup was heparan sulphate glycosaminoglycan biosynthesis signalling pathways. **D** The main enrichment analysis result in the high ICI score subgroup was apoptosis, cytokine receptor interaction, toll-like receptor and leucocyte transendothelial migration signalling pathways. **E** Association between different ICI score groups and overall survival in the TARGET and GSE85047 cohorts. Log rank test, *p* = 0.028

We performed principal component analysis (PCA) to calculate the quantitative indicators of different ICI subtypes, which were labelled as ICI scores A and B. Then, we obtained a prognosis-related score defined as the ICI score and divided the patients in the TARGET and GSE85047 cohorts into high and low ICI score subgroups. The distribution of different patients in diverse gene clusters was summarized in an alluvial graph (Fig. 4A). The Gene ICI Cluster A samples that exhibited higher ICI scores were significantly associated with an improved survival outcome of 72.3% (136/188), while the samples in the gene ICI Cluster B exhibited lower ICI scores and a lower overall survival outcome (58.2%, 131/225). Then, we analysed the relationship between the immune tolerance of NB patients and the prognostic value of the ICI score. A few representative immune activity-related targets (such as PD-L1, CTLA4, LAG3, IDO1 and HAVCR2) and immune checkpoint-related targets were selected [26, 27]. In our study, except for LAG3, all selected immune activity-related targets and immune checkpoint-related targets were remarkably overexpressed in the high ICI group. Gene set enrichment analysis (GSEA) showed that the heparan sulphate glycosaminoglycan biosynthesis signalling pathway was associated with the low ICI score group, while apoptosis, cytokine receptor interaction, toll-like receptor and leucocyte transendothelial migration signalling pathways were associated with the high ICI score group (Fig. 4C and D). The Kaplan–Meier analysis revealed that the samples in the high ICI score subtype were significantly associated with better outcome, while samples in the low ICI score subtype had an unfavourable OS (*p* = 0.025; Fig. 4E).

## Discussion

Neuroblastoma (NB) is the most common extracranial solid tumour in children and is an important cause of childhood death from cancer. Patients in the low-risk groups responded better to surgery and chemotherapy. However, high-risk patient groups often have extensive metastases. Even with high-intensity chemotherapy combined with surgery, radiotherapy, and autologous bone marrow stem cell transplantation, the rate of long-term survival in high-risk groups is still less than 50%. High-risk NB has a high degree of malignancy and is resistant to radiotherapy and chemotherapy. With advanced knowledge of the immune system and tumour microenvironment, new targets may be identified that facilitate immunotherapy treatment for NB patients.

Immunotherapy has shown encouraging results and may improve the survival status and quality of life for high-risk NB patients [13, 14]. GD2 and B7H3 chimeric antigen receptor (CAR) T cells maintained high metabolic fitness comparable to resting T cells, were highly resistant to exhaustion and have great in vivo efficacy [28]. Using of the oncolytic viruses along with Ganglioside GD2-specific CAR-T cells to treat the neuroblastoma led to an increase in the attraction and survival of the CAR-T cells [29]. However, the inconsistency of the results of previous exploratory studies of tumour surface markers highlights the necessity of determining the ideal subgroup of NB patients for immunotherapy, which is still a major challenge in immunotherapy. A monoclonal antibody named dinutuximab attached to GD2 on the surface of neuroblastoma cells has been used as an immunotherapy drug for neuroblastoma [6]. Recently, various lines of evidence have confirmed the potential of PD-1/PD-L1 in immunotherapy for neuroblastoma [30, 31]. However, only a small percentage of patients benefit from immunotherapy. Therefore, it is important to identify subgroups that can benefit from immunotherapy. In our research, we validated a method that can be used to quantitatively assess the integrated tumour microenvironment in NB. Our results indicated that the ICI score was not only an effective prognostic feature but also a useful identifier of NB patients who could benefit from immunotherapy.

As in previous studies, patients with specific active immunity have a significantly better prognosis, while high-risk NB patients with immune cell dysfunction have increased immune resistance, resulting in tumour progression and unfavourable prognosis [32, 33].

Here, we found that the provided ICI score was suitable for NB and had the potential to be a specific prognostic biomarker. First, we analysed the ICI from 438 NB sample cohorts in two databases and divided NB into two different immune subtypes. The results showed that the relative content of resting dendritic cells in all samples was nearly zero. The ICI Cluster A samples were marked by a high ImmuneScore and high levels of naive B cells, regulatory T cells (Tregs), naive CD4 T cells and CD8 T cells. The samples in ICI Cluster B exhibited a significantly higher StromalScore and levels of resting memory CD4 T cells, M0 and M2 macrophages and resting mast cells. However, the Kaplan–Meier survival curve results were not statistically significant.

Studies have shown that a number of cytokines and TME components and the host’s immune response have a positive impact on the antitumor response and maintain a dynamic balance. These molecular changes during tumorigenesis may interfere with the function of infiltrating immune cells, resulting in the destruction of the dynamic balance between immune tolerance and activity [34].

We utilized the combination of ICI and immune-associated gene expression as a new method for patient-specific customized treatment strategies. Then, the novel immune-related genes identified based on the significant ICI gene clusters were used to explore their prognostic significance by integrating ICI gene clusters with survival information. The Kaplan–Meier analysis revealed that the samples in gene Cluster A were significantly associated with improved outcome, while samples in the remaining gene clusters were associated with a poor prognosis (log rank test, *p* = 0.040; Fig. 2E). Patients with the lowest immune score and matrix score values were grouped into the ICI gene Cluster B subtype, which is associated with the immune cold phenotype. Patients with ICI gene Cluster A and C subtypes showed higher inflammatory cell density and immune scores. Moreover, we found that the increased levels of M2 macrophage infiltration in the ICI gene Cluster C subtype was related to a high stromal score, indicating an unfavourable prognosis [25]. Samples of the ICI gene Cluster A subtype were associated with a good prognosis and patients in this group could benefit from immunotherapy, unlike those of the other groups.

There is an urgent need to quantitatively assess the ICI pattern of neuroblastoma because of the heterogeneity of the individual microenvironment. Individual models have been well established in colorectal cancer, head and neck squamous cell carcinoma and breast cancer to improve prediction accuracy [18, 35, 36].

The potential signature subtypes were established after quantitatively assessing the ICI model in our study. The Kaplan–Meier analysis showed that the high ICI score subtype was significantly associated with better outcome, while samples in the low ICI score subtype had an unfavourable OS (log rank test, *p* = 0.025). The highest number of immune checkpoint-associated targets and immune activity-associated targets that were overexpressed occurred in the high ICI subgroup. It is inferred that patients with high ICI NB subtypes were more likely to respond to immunotherapy than those in other groups were. For example, PD-1/PD-L1-targeted immunotherapy was more likely to be effective because PD-L1 expression was more highly expressed in the high ICI NB subgroup. The genes of the immunosuppressive heparan sulphate glycosaminoglycan biosynthesis signalling pathway were remarkably enriched in the low ICI score subgroup according to GSEA. Destroyed integrity of the extracellular matrix and basement membrane is the primary condition for the invasion and metastasis of malignant tumours. Microscopic damage to the extracellular matrix in the tumour microenvironment is a sign of aggressive tumour growth [37]. Heparanase (HPSE), as an endogenous endoglycosidase, is highly expressed in high-risk neuroblastoma. Tumour cells secrete a large amount of heparanase to destroy the strong network structure of the extracellular matrix and basement membrane, promoting the invasion of inflammatory cells and metastasis of tumour cells and the progression of related diseases [38]. In the high ICI group, apoptosis, cytokine receptor interaction, Toll-like receptor and leucocyte transendothelial migration signalling pathways were enriched.

## Conclusion

In summary, it is urgent to constantly explore novel prognostic markers for the treatment of tumours. We found that the ICI score presented here is applicable in NB and can also be used for the development of specific prognostic biomarkers. We found that differences in ICI subtypes were related to individual heterogeneity and treatment efficacy. In addition, this study can help develop new ideas for NB immunotherapy methods.

## Data Availability

The data used to support the findings of this study are included within the article.

## Abbreviations

TME: Tumour microenvironment
NB: Neuroblastoma
ICI: Immune cell infiltration
OS: Overall survival
GD2: Disialoganglioside
PD-1: Programmed death 1
PD-L1: Programmed death ligand 1
NGS: Next-generation sequencing
DEGs: Differentially expressed genes
PCA: Principal component analysis
